# Short-Term Exposure to Tobacco Toxins Alters Expression of Multiple Proliferation Gene Markers in Primary Human Bronchial Epithelial Cell Cultures

**DOI:** 10.1155/2011/208563

**Published:** 2011-04-05

**Authors:** Imran S. Chaudhry, Ashraf El-Meanawy, Amer Khiyami, Joseph F. Tomashefski, Rhoderick N. Machekano, Lawrence Kass

**Affiliations:** ^1^Department of Pathology, MetroHealth Medical Center, Case Western Reserve University, Cleveland, OH 44109, USA; ^2^DynaLIFE Dx, Suite 200, 10150 102 Street, Edmonton, AB, Canada T5J 5E2; ^3^Department of Medicine, MetroHealth Medical Center, Case Western Reserve University, Cleveland, OH 44109, USA; ^4^Kidney Disease Center, Medical College of Wisconsin, Milwaukee, WI 53226, USA; ^5^Center for Health Care Research and Policy, MetroHealth Medical Center, Case Western Reserve University, Cleveland, OH 44109, USA

## Abstract

The biological effects of only a finite number of tobacco toxins have been studied. Here, we describe exposure of cultures of human bronchial epithelial cells to low concentrations of tobacco carcinogens: nickel sulphate, benzo(b)fluoranthene, N-nitrosodiethylamine, and 4-(methylnitrosamino)-1-(3-pyridyl)-1-butanone (NNK). After a 24-hour exposure, *EGFR* was expressed in cell membrane and cytoplasm, *BCL-2* was expressed only in the irregular nuclei of large atypical cells, *MKI67* was expressed in nuclei with no staining in larger cells, cytoplasmic *BIRC5* with stronger nuclear staining was seen in large atypical cells, and nuclear *TP53* was strongly expressed in all cells. After only a 24-hour exposure, cells exhibited atypical nuclear and cytoplasmic features. After a 48-hour exposure, *EGFR* staining was localized to the nucleus, *BCL-2* was slightly decreased in intensity, *BIRC5* was localized to the cytoplasm, and *TP53* staining was increased in small and large cells. *BCL2L1* was expressed in both the cytoplasm and nuclei of cells at 24- and 48-hour exposures. We illustrate that short-termexposure of a bronchial epithelial cell line to smoking-equivalent concentrations of tobacco carcinogens alters the expression of key proliferation regulatory genes, *EGFR, BCL-2, BCL2L1, BIRC5, TP53*, and *MKI67*, similar to that reported in biopsy specimens of pulmonary epithelium described to be preneoplastic lesions.

## 1. Background

Cigarette smoking is a major cause of lung cancer. 90% of male and 75–80% of female lung cancer deaths in the USA are smoking related [1]. As defined by the International Agency for Research on Cancer (IARC), each cigarette contains a mixture of more than 60 known carcinogens [2]. At least twenty of these carcinogens have been linked to tumors [1]. 

Bronchial epithelium undergoes a stepwise preneoplastic process encompassing various morphological and molecular changes before overt development of lung cancer [3]. The 5-year survival for patients with lung cancer is approximately 15% [4], and patients with nonsmall cell lung cancer in stage I-A disease have a 33% chance of recurrence within 5 years after complete surgical resection [5, 6]. Currently, there is no immunohistochemical or morphological marker, available for metaplasia, dysplasia, or carcinoma insitu, which reliably predicts the biological behavior of preneoplastic lesions. 

The *BCL-2* [7–9] and *BCL2L1* antiapoptotic genes are expected to contribute to carcinogenesis. *BCL-2* prolongs survival of noncycling cells and inhibits apoptosis of cycling cells [10, 11]. Epidermal growth factor receptor (*EGFR*) is a tyrosine kinase receptor which increased cell proliferation [12–16]. *BIRC5* is a member of the inhibitor of apoptosis protein (IAP) family, a cell-cycle-regulated bifunctional protein expressed in G2/M phase [17–19]. *BIRC5* may overcome G2/M phase checkpoints to enforce progression of cells through mitosis, favoring development of neoplastic clones. *MKI67* is expressed during all active phases of the cell cycle. The fraction of *MKI67*-positive tumor cells (*MKI67* labeling index) provides correlation with the clinical course of disease [20–22].

While malignant transformation can be induced in bronchial epithelial cell cultures, the effects of exposure to individual tobacco carcinogens have not been well studied during phases preceding the development of overt cancer. The purpose of our current work is to study the effect of individual tobacco carcinogens on cultured human bronchial epithelial cells. 

## 2. Materials and Methods

### 2.1. Reagents

Sources of the toxins, cell line, antibodies, and reagents used in this work are listed in supplementary data (see the Supplementary Material available online at doi:10.1155/2011/208563). 

### 2.2. Cell Culture

Bronchial epithelial growth medium (BEGM) was prepared as previously described [23]. The cryopreserved cell line was thawed and initially grown in 35 mm plastic dishes in the above-specified medium in a humidified 5% CO_2_ incubator at 37°C until cells were 70–80% confluent. Cells were lifted by trypsinization and replated in 24-well plates containing glass cover slips until they are 70–80% confluent. 

### 2.3. Toxin Exposure

Toxin solutions (Table 1) were evaluated for effects on nonspecific esterase (NSE) and cytomorphology assessed by PAP staining and phase contrast microscopy. We evaluated the effects of nickel sulphate (heavy metal), benzo(b)fluoranthene (polyaromatic hydrocarbon), N-nitrosodiethylamine (a tobacco nitrosamine), and NNK (a nicotine derivative) using electron microscopy and immunohistochemistry after 24 and 48 hours of exposure at low carcinogen concentration. The final concentration of solvents in the culture media was less than 0.01% as previously described [24]. The working concentration of toxins were based on the epithelial exposure to toxin typically present in one cigarette [25]. The median concentration of each toxin in one smoked cigarette was taken as the medium concentration (M), and lower (L) and higher (H) dose exposure concentrations were arbitrarily determined (Table 1). Two controls were included with each carcinogen exposure, a “solvent control” (S) corresponding to the solvent used to dissolve the toxin (used equivalent to the highest concentration) and a “negative control” (N) containing only the growth medium. Cells were incubated in the culture media containing toxins or controls for 24 and 48 hours. Cells were then washed with DPBS. For Pap staining, cells were fixed in 95% alcohol for 30 minutes, air-dried, and stored at 4°C. For electron microscopy, cells were trypsinized, washed twice in growth medium and, centrifuged. The cell pellet was fixed in 2.5% glutaraldehyde and refrigerated at 4°C. 

### 2.4. Nonspecific Esterase (NSE) Cytochemistry and Papanicolaou (PAP) Staining

We used NSE staining as a measure of cell activity and to determine the minimally toxic concentration of tested chemicals that is capable of inducing a meaningful change in the cytomorphology. The staining was done as previously described [26, 27]. The stained cells on the cover slips were air-dried and mounted inverted on glass slides using Permount mounting media (Fisher Scientific, Pittsburgh, PA). 

### 2.5. Immunohistochemistry (IHC)

Fixed cytospin slides were incubated in 3% hydrogen peroxide for 15 minutes at room temperature, rinsed in distilled water, incubated for 10–15 minutes at room temperature with blocking serum, followed by the application of primary mouse monoclonal antibodies for *EGFR* (1 : 100), *TP53* (1 : 100), *BIRC5* (1 : 150), *MKI67* (1 : 50), *BCL-2* (1 : 100), and *BCL2L1* (1 : 150). The primary antibodies were incubated for 60 minutes at room temperature. The slides were washed three times with PBS 0.2% Tween followed by application of biotin-labeled antimouse IgG and further incubated for 30 minutes at room temperature. The cells were then washed with PBS 0.2% Tween, and a working dilution of fresh DAB solution was added. Slides were counterstained in fresh Gill's hematoxylin, placed in ammonia water for 5–10 seconds, dehydrated, and mounted with Permount.

### 2.6. Electron Microscopy (EM)

Standard tissue processing for electron microscopy was used [28]. Toxin-treated glutaraldehyde-fixed cell pellets ware washed in Millonig's phosphate buffer and placed in 1% osmium tetroxide for one hour, washed twice with Millonigs buffer, dehydrated by passing through graded ethanol twice, 15 minutes each, and finally left in 100% ethanol for 30 minutes. Then, the pellet was treated with 1 : 1 and 3 : 1 working Spurr : ethanol mixtures followed by a 100% working Spurr solution, 30 minutes each, and embedded in the center of a beam capsule. Thick and thin sections were prepared and examined with a transmission electron microscope (Tecnai 12 T; FEI Company, Hillsboro, Oregon). 

### 2.7. Phase Contrast Microscopy

All slides after defined exposure times and prior to any further processing were viewed using an inverted phase contrast microscope (Leica DMIRE2, Germany) at 20x, and appropriate images were taken. Regular digital images of stained slides were taken at 60x for morphometry using a microscope (Olympus BX51, Center Valley, PA) fitted with a digital camera (Olympus DP71, Center Valley, PA). A bar of 100 microns was drawn using an internal scale at 60x, later used to calibrate ImageJ software. 

### 2.8. Evaluation of NSE, ki-67, p53 Staining, and Morphometry

NSE staining was evaluated in 100 cells under 60x objective. Negative cells were graded as zero, minimal staining and/or forming an incomplete rim around the nucleus whether punctate or homogeneous as 1, moderate staining and/or forming a complete rim around the nucleus whether punctate or homogeneous as 2, and strong staining forming a complete rim around the nucleus as 3. Some small pyknotic nonstaining cells, most probably representing basal cells, identified both in the unexposed and exposed cultures were not counted. For morphometry of cytological effects, nuclear:cytoplasmic ratios were determined with NIH ImageJ software. Manual threshold of an 8-bit greyscale image was performed and regions of interest were selected with the “region of interest” (ROI) manager. A line-drawing tool was used to select areas not amenable to thresholding. ImageJ was calibrated in a set scale window by using the 100 *μ*m scale bar captured earlier at 60x magnification. For density calibration, ImageJ was calibrated following the procedure listed at the HIN ImageJ manual (http://rsb.info.nih.gov/ij/). Biostatistical analysis and graphical displays were done using R software (http://www.r-project.org/).

## 3. Results

### 3.1. NSE, PAP Staining, and Morphometry

**Figure 1:**
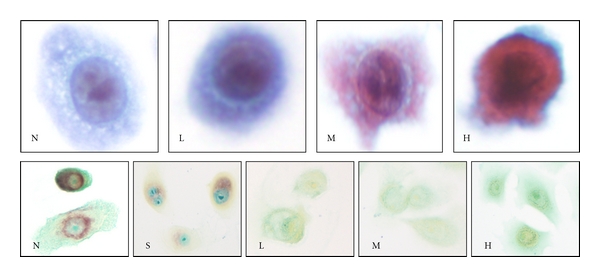
(60x magnification) Top Panel: effects of benzo(b)fluoranthene on morphology assessed by Papanicolaou stain. Note changes in cell size, nuclear size, nucleolar size, and nuclear and cytoplasmic density. Bottom Panel: histochemical staining for nonspecific esterase (NSE) of control and benzo(b)fluoranthene-treated cells. The negative (N) and solvent (S) controls (acetone exposure) display positive perinuclear, punctuate, or Golgi pattern of dense NSE staining while benzo(b)fluoranthene-exposed cells showed completely negative staining at all exposure concentrations. N: negative control, S: solvent control, L: low carcinogen concentration, M: medium carcinogen concentration, and H: high carcinogen concentration.

The morphology of untreated cells was used as the basis for comparison. These cells displayed regular and smooth cellular and nuclear contours and small nucleoli. All carcinogen-treated cells displayed a range of morphologic changes; a representative image depicting benzo(b)fluoranthene-treated cells is displayed in Figure 1 (top panel). At low concentration, cells displayed nuclear enlargement, minimal to slight nuclear contour irregularities, and enlargement of the nucleolus with minimal changes in cell membrane outline. Additionally, at medium concentration, cells demonstrated a mild to moderate increase in nuclear density, a further increase in nucleolar size, shrinking of cytoplasmic membranes, changes in cell size, and nuclear:cytoplasmic (N : C) ratio. At the highest concentration, nuclear hyperchromasia increased significantly with marked nuclear contour irregularities and inconspicuous nucleoli. A second population of large atypical cells with irregular and folded nuclei emerged in the exposed cultures constituting only 5–10% of the total population. These large cell population were excluded from the morphometric evaluation which showed that the N : C ratios for seven of the carcinogen exposures (nickel sulphate, chromium chloride, benzo(b)fluoranthene, indeno(1,2,3,-cd)pyrene, ethyl carbamate, N-nitrosodiethylamine, and NNK) were statistically significantly higher than controls at different carcinogen concentrations (Table 2). PAP-stained, vehicle-treated cells did not show any of the morphological changes described above (not shown). NSE staining was consistently absent in all twelve toxin-exposed cell groups at all concentrations with the exception of cadmium and chromium chloride, which showed weak activity at low (L) concentration. Intense peroxidase staining was observed in medium only control cells (N). Cells incubated with solvents (S) demonstrated NSE activity comparable to medium only controls (Figure 1). 

### 3.2. Phase Contrast Microscopy and Electron Microscopy

**Figure 2:**
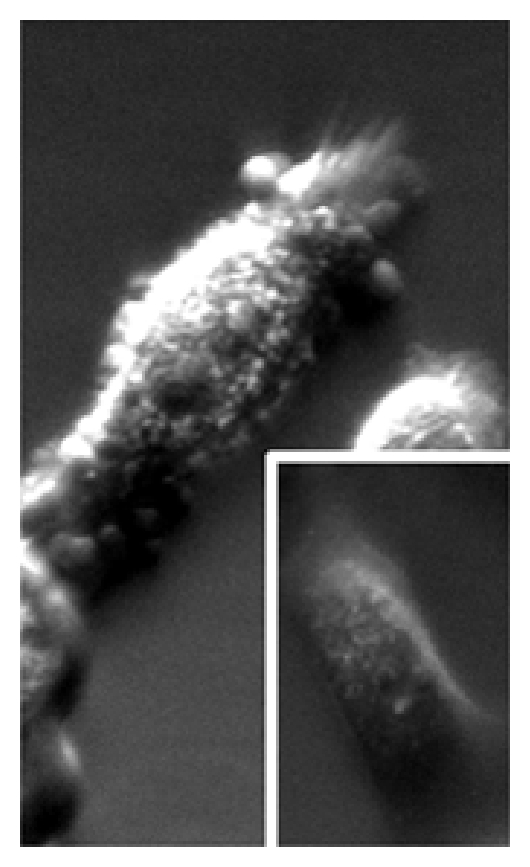
Although seen with many carcinogen exposures as occasional finding at higher concentrations, striking blebbing of the cytoplasmic membrane was noted with 24-hour N-nitrosodiethylamine exposure at extremely low concentration (1 ng/mL) detected by phase contrast microscopy at 60x. *Inset:* negative control as comparison with smooth and regular cell surface.

**Figure 3:**
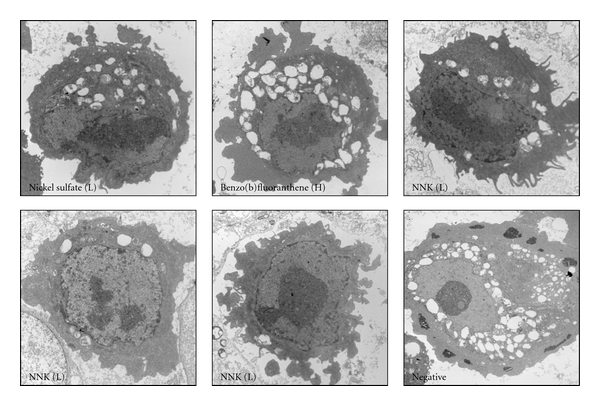
Electron microscopy shows striking and consistent nuclear and nucleolar changes with markedly increased size and changes in shape including elongation and multiple enlarged and/or irregularly shaped nucleoli and nucleolus appearing to span the inner diameter of the nucleus. The electron dense cytoplasmic granular material is present only in the negative control. NNK is represented in multiple images to show the pleomorphism in the toxin-induced nuclear changes. Direct magnification: 6500x, Print magnification: 11200x at 7 inch.

All toxin-treated cell groups demonstrated shrinkage, small cell size, and nuclear granularity with membrane irregularity using phase contrast microscopy. A notable finding was cytoplasmic blebbing and outpouching with loss of cell membrane smoothness induced by N-nitrosodiethylamine even at low concentration (Figure 2). Cells treated with nickel sulphate, benzo(b)fluoranthene, and NNK revealed striking and consistent changes in the nucleoli by electron microscopy. Nucleolar size increased markedly with changes in shape including elongation, as well as multiple enlarged and/or irregularly shaped nucleoli. In some cells, the nucleolus appeared to span the inner diameter of the nucleus. Electron dense nonmembrane bound granular material was occasionally noted in the cytoplasm of cells not treated with toxins (Figure 3). 

### 3.3. Immunohistochemistry

**Figure 4:**
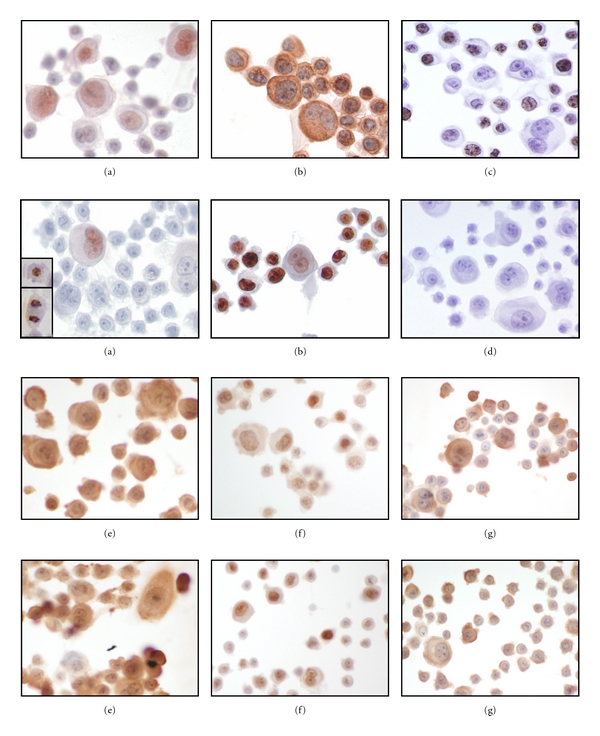
Immunostaining for *BCL-2*, *EGFR, Ki-67*, *BCL*-XL, p53, and BIRC5 after 24-hr (rows 1 and 3) and 48-hr (rows 2 and 4) exposure to benzo(b)fluoranthene at low concentration. Original magnification 60x. (a) *BCL-*2. Only nuclei of large atypical cells stain positively for *BCL*-2 at 24-hour exposure; staining is decreased slightly at 48-hour exposure. *Inset:* A pyknotic cell (top) and mitotic figure (bottom) stain strongly for Bcl-2. (b) *EGFR*. *EGFR* localized to cell membrane, cytoplasm, and weakly to nuclei of all cells at 24-hour exposure; staining shifts to the nuclei at 48-hour exposure, strongly in small cells and weakly in large atypical cells. (c) *MKI67*, assessed at 24-hr exposure only, strongly stained small cell nuclei with negative staining in large cells. (d) Negative control. Representative image of absence of immunoperoxidase staining. (e) *BCL2L1* Similar cytoplasmic and nuclear staining at both 24- and 48-hour exposure. F. *TP53*. Nuclear staining is noted in nearly all cells at 24-hour exposure, with increased intensity at 48-hour exposure. (g) *BIRC5* staining is localized mainly in the cytoplasm of cells following the 24-hour exposure with relatively stronger nuclear staining in large atypical cells. At 48-hour exposure, only cytoplasmic staining is observed with focal membranous staining.

Negative control cells (media only or solvent) showed absent staining for all antibodies except membrane staining for *EGFR* (Figure 4). By omitting the primary antibody, “negative immunostaining controls” were also evaluated, none of which showed any staining (not shown). *BCL-2* staining at 24-hr exposure was negative in all cells except large atypical cells with multiple and irregular nuclei showing *BCL-2* localized to their nuclei with weak to moderate cytoplasmic staining. Cells with pyknotic and shrunken nuclei and those undergoing mitosis demonstrated strong nuclear positivity. These changes were consistent among all four toxins tested at both 24 and 48 hours; however, a few large atypical cells were positive at 48 hrs for *BCL-2*. *MKI67* was not evaluated at 48 hours. At 24-hour exposure, *MKI67* showed high reactivity in a speckled and granular pattern outlining the chromatin. In stark contrast to *BCL-2*, *MKI67* did not stain large atypical cells with irregular and multiple nuclei. Twenty large atypical cell nuclei and 100 small-cell nuclei were counted three times and the average number of nuclei which were positively stained for *MKI67* was represented as a percentage of the total (Table 3). A similar approach was used to assess nuclear *TP53* staining in both small and large atypical cells (Table 4). Small cells stained positive while large cells were predominantly negative. In cells exposed to benzo(b)fluoranthene the number of *TP53* positive cells increased from 68% to 82.6% at the 24- versus 48-hour exposure interval. With N-nitrosodiethylamine, immunostaining for *TP53* was positive in a greater proportion of cells (82.3%) at 24 hours (82.3% versus 64.6%). Staining pattern of *TP53* remained the same at 24- and 48-hour exposures in the large atypical cells. At 24 hour exposure, *EGFR* demonstrated strong cytoplasmic and cell membrane staining and very weak nuclear staining in all cells. At 48-hour exposure, only nuclei of small cells stained strongly positive with no cytoplasmic or cell membrane staining. The large atypical cells with multiple irregular nuclei were mostly negative with occasional cells demonstrating weak and variable *EGFR* staining in the cytoplasm and/or nucleus. For all four toxins tested at 24- and 48-hour exposures, *BIRC5* activity was distributed primarily in the cytoplasm of cells, although some nuclear staining was also observed in cells exposed to nickel sulphate. Relatively stronger nuclear staining for *BIRC5* was noted in large atypical cells with irregular nuclei. At the 48-hour exposure, only cytoplasmic staining was observed in small and large cells. Some membranous staining in cells exposed to benzo(b)fluoranthene was noted. *BCL2L1* staining was present in both the cytoplasm and nucleus of the cells at 24- and 48-hour exposure. *BCL2L1* was more strongly positive in benzo(b)fluoranthene compared to nickel sulphate exposed cells at 24 hours (Figure 4). In general, there was no difference in cell viability between toxin treated cells and control cells (data not shown).

## 4. Discussion

In this study we have developed a reproducible technique for exposing human bronchial epithelial cells in culture to soluble tobacco toxins and have observed the early effects of these toxins on cell morphology, NSE activity, and selected gene expression. Although the immunohistochemical profile of various proteins in invasive lung carcinomas has been extensively studied, there are relatively few studies of protein expression in precancerous lesions [29], and virtually no information on the changes in cultured bronchial epithelial cells after toxin exposure. Among tobacco toxins benzo(a)pyrene (BaP), a prototypic polyaromatic hydrocarbon (PAH), has perhaps been the most extensively studied. The unique effects of other toxins are less well recognized in the complex composite milieu of tobacco smoke exposure experiments. There is limited data on exposure levels of individual tobacco toxins [30–36]. Accordingly, we chose a range of exposure concentrations based on the reported concentration of toxins found in a smoked cigarette [25]. In our experiments, cells were exposed for 24 and 48 hours, allowing adequate time for protein synthesis. Dibenz(a,h)anthracene and benzo(k)fluoranthene, which were soluble only in toluene, were excluded from our analysis to avoid the confounding issue of toluene cytotoxicity. 

After exposure to toxins, cells displayed a spectrum of morphologic changes including nuclear enlargement and contour irregularities, enlargement of nucleoli, increase in nuclear density, shrinking of cytoplasmic membrane, changes in cell size, and N : C ratio, progressively accentuating from low to higher concentrations. In the exposed cell cultures, there emerged a second population of abnormal large atypical cells with very irregular and folded nuclei constituting 5–10% of the cellular population. All these features resemble those which characterize dysplastic cells. Our results further indicate a consistent decrease in NSE in toxin-treated cells relative to the controls for all tobacco toxins. 

*EGFR* is overexpressed in human cancer cells and is linked to metastasis and resistance to treatment. In our study, *EGFR* was strongly positive in the cell membrane and cytoplasm with only weak nuclear staining following a 24-hour toxin exposure. After 48 hours of exposure, all *EGFR* staining was concentrated in the nucleus. The nonexposed cells showed only indistinct membranous rim-like staining. Immunostaining for *EGFR* has been shown to increase with the severity of dysplasia in preneoplastic and early neoplastic bronchial epithelium. Conversely, decreased expression of *EGFR* follows regression of bronchial squamous metaplasia [13, 16, 19, 29]. The nuclear shift of *EGFR* after a 48-hour toxin exposure correlates with the observation that, in response to growth factor stimulation, a fraction of *EGFR* moves from the cell surface to the nucleus, possibly interacting with STAT3 and directly regulating gene expression [15]. 

*BCL-2* expression has been seen in less aggressive tumor behavior and is linked to increased cell survival [7]. Our results indicate absent immunostaining for *BCL-2* in the majority of cells exposed to toxins for 24 or 48 hours. However, the subpopulation of large atypical cells with irregular and large nuclei demonstrates positive nuclear and weak cytoplasmic *BCL-2* staining at 24 hours which persisted with decreased intensity at 48-hrs. In the unexposed cells, *BCL-2* staining was undetectable. Reported studies in tissue sections show basal *BCL-2* staining in normal epithelium, basal and suprabasal staining in metaplastic epithelium, and increasingly aberrant *BCL-2* expression with increasing grades of dysplasia [7–9]. Substantial pools of *BCL-2* have been identified within interphase nuclei controlling cellular proliferation that may induce rather than protect cells from apoptosis [8, 37]. Our results also suggest that *BCL-2* is expressed early after toxin exposure. 

The fact that *MKI67* is present during all active phases of the cell cycle and undetectable in resting cells makes it an excellent marker for determining the growth fraction of a given cell population [22]. We found high *MKI67* immunostaining both speckled and granular patterns, outlining the nuclear chromatin material of toxin-exposed cells (Table 3). In contrast to *BCL-2*, immunostaining for *MKI67* was not seen in the population of large atypical cells with multiple or irregular nuclei. *MKI67* was completely absent in all cells in the unexposed cultures. Various studies have reported high *MKI67* activity in bronchial dysplastic lesions including 62.5–100% by Wang et al. [36], 1 to 60% by Tan et al. [20, 36], and 49% expression in small cell lung cancer specimens by Paik et al. [36, 38]. Our finding of high *MKI67* immunostaining in toxin-exposed cells is therefore similar to observations reported in tissue sections from dysplastic lesions.

Studies have reported negative *BIRC5* staining in normal bronchial epithelium, minimally atypical hyperplastic, and nonneoplastic lesions adjacent to tumors. Both nuclear and/or cytoplasmic *BIRC5* expression has been identified in metaplastic, dysplastic, and hyperplastic lesions with moderate dysplasia [18, 36]. The level of *BIRC5* correlates with the degree of dysplasia and is highest in carcinomas [19, 36]. *BIRC5* was found to be localized to the nucleus in 70% of early NSCLC's and both in the cytoplasm and nucleus in 54% of cases. Moreover, it was also identified in atypical mitotic figures and in giant multilobed neoplastic nuclei [39]. We observed *BIRC5* mainly in the cytoplasm of cells following the 24-hour exposure, with some nuclear staining in cells exposed to nickel sulphate and relatively stronger nuclear *BIRC5* in large atypical cells with irregular nuclei. After 48-hour exposure, only cytoplasmic staining was observed, with focal membranous staining in cells exposed to benzo(b)fluoranthene. This pattern in cultured bronchial cells of persistent cytoplasmic *BIRC5* immuno-reactivity and minimal nuclear reactivity after 48 hours of exposure to toxins somewhat differs from other tissue-based studies in which a predominance of nuclear immuno-reactivity has been reported. This discrepancy may relate to the short time interval of toxin exposure in our cell culture model or to the combination effect of multitoxin exposure in previous studies. 

In our study, strong *TP53* nuclear staining was noted in the cells exposed to some toxins for 24 hours which further increased after 48 hours (Table 4). The variation of response kinetics to different toxins could be a reflection of a different mechanism of stimulating *TP53*. Altering signaling pathway through protein phosphorylation or direct modification of intermediate signaling compounds is usually very fast. On the other hand, alteration of mRNA expression or stability takes a longer time, albeit shorter than an epigenetic modification. In nature, the half-life of these compounds is long (2–>300 days). However, the half-life of these compounds was never examined in tissue culture. In the control cultures, only rare small cells showed positive *TP53* staining. Several studies demonstrate suppression of *BIRC5* by *TP53* [17]. The *TP53* immunostaining is reported to be infrequent in normal or metaplastic mucosa but may be seen in as many as 30% of cases of mild bronchial epithelial dysplasia [36]. Progressively increased suprabasal expression of *TP53* can be seen with increasing grades of dysplasia. The likelihood of invasive cancer has been positively correlated with the degree of *TP53* expression in bronchial epithelium from the same lung lobe [40], suggesting that *TP53* may have predictive value in assessing the biological behavior of preneoplastic endobronchial lesions. In another study, 41% of patients with dysplastic lesions showing >10% *TP53*-positive nuclei later developed lung cancer whereas only 23% of those with *TP53*-negative lesions progressed to cancer (positive and negative predictive value of 78% and 77%, resp.) [41]. Our results show that exposure to tobacco toxins results in appearance of significant *TP53* nuclear immuno-reactivity compared with unexposed (control) cells, and that the intensity of staining increases with the duration of exposure to tobacco toxins.

There is little information in the literature on the expression of *BCL2L1* in preneoplastic/dysplastic pulmonary lesions. Cytoplasmic expression of *BCL2L1* and elevated *BCL2L1* gene transcripts have been reported in 81.7% and 60% of lung cancers, respectively. Patients with tumors expressing *BCL2L1* showed shorter median survival compared to patients without *BCL2L1*-expressing tumors [11]. Either cytoplasmic or nuclear expression of *BCL2L1* was found in 81.5% and 30.4% of lung cancers, respectively. Nuclear *BCL2L1* expression correlated among all histologic types with TNM stage IV and the high expression of cytoplasmic *BCL2L1* (81.9%) in resected non-small cell lung cancers without any apparent influence on clinical outcome [10]. In our study, both cytoplasmic and nuclear *BCL2L1* staining was present at 24- and 48-hour exposure intervals. *BCL2L1* was more strongly positive in benzo(b)fluoranthene, exposed cells compared to nickel-sulphate-treated cells at 24 hours, suggesting that *BCL2L1* expression may be toxin dependent.

In summary, our studies describe the effects of known pulmonary tobacco toxins on an established cell line of human bronchial epithelial cells. Although the concentration of these toxins in cigarette smoke has been determined, we could only estimate the appropriate concentration for application to cells in tissue culture. Under the conditions of our experiments, we found that a brief exposure of cells to tobacco toxins produces consistent and reproducible morphologic changes of cell and nuclear size and shape. Using immunohistochemistry we found that cells treated with toxins showed emergence of activities of *EGFR*, *BCL-2*, *MKI67*, *BCL2L1*, *BIRC5,* and *TP53* not found in untreated control cells. These changes are similar to those reported in tissue specimens of preneoplastic lesions and fully developed lung cancer. The findings of this study suggest that changes in expression of these proteins occur at a rapid rate after exposure of the cells to toxins, raising the possibility that some changes associated with overt malignancy might occur rapidly *in vivo* following toxin exposure. Prolonged or intermittent toxin exposure effect on cells is not known. Additional studies using human bronchial epithelial cell lines with other toxins or chronic intermittent exposure might increase our understanding of pathways involved in the development of lung cancer.

## Supplementary Material

Supplemental Materials: The supplemental materials include the source of the chemical reagents, cell line, and antibodies used in this study.

## Figures and Tables

**Table 1:** Tobacco carcinogens and dilutions used.

No.	Tobacco carcinogens	Toxin range: ng/cigarette	Working concentrations			Solvent used for stock soln.
			High ng/ml	Med^#^ng/ml	Lowng/ml	
(1)	Nickel Sulphate**	0–510	800	500	200	Water
(2)	Cadmium Chloride	0–6670	1200	700	200	Water
(3)	Chromium Chloride	0.2–500	800	500	200	Water
(4)	Sodium Selenite	0–1400	1900	1400	900	Water
(5)	Benzo(b)fluoranthene**	4–22	44	22	2	Acetone
(6)	Indeno(1,2,3,-d)pyrene	4–20	40	20	2	ETOH
(7)	Ethyl Carbamate	20–38	80	40	2	ETOH
(8)	Dibenz(a,h)anthracene*	4	12	4	1	Toluene
(9)	N-Nitrosodiethylamine**	0–2.8	9	3	1	ETOH
(10)	5-Methylchrysene	0.6	3	1	0.25	ETOH
(11)	Dibenzopyrene	1.7–3.2	9	3	1	ETOH
(12)	Dibenz(a,h)acridine	0.1	3	0.1	0.25	Acetone
(13)	NNK**	80–770	1300	800	300	Water
(14)	Benzo(k)fluoranthene*	6–12	24	12	2	Toluene

*Excluded from morphometry and immunohistochemical analysis due to cytotoxicity.

**Used in immunohistochemistry only.

^#^Equivalent volume of solvent/mL BEGM used as solvent (S) control in NSE staining only.

**Table 2:** N : C ratios at 24-hour carcinogen exposure compared to vehicle controls.

Carcinogen	N : C ratio at high dose	N : C ratio at med dose	N : C ratio at low dose
Nickel sulphate	0.338 (0.033)	0.318 (0.032)	0.196 (0.034)
	*P* = .002	*P* = .017	*P* = .042
Chromium chloride	0.300 (0.031)	0.365 (0.033)	0.370 (0.033)
	NS	*P* < .05	*P* < .05
Benzo(b)fluoranthene	0.360 (0.034)	0.242 (0.033)	0.253 (0.033)
	*P* < .05	NS	NS
Indeno(1,2,3,-cd)pyrene	0.184 (0.046)	0.248 (0.033)	0.209 (0.034)
	*P* = .03	NS	NS
Ethyl carbamate	0.254 (0.029)	0.273 (0.028)	0.254 (0.028)
	NS	NS	NS
N-Nitrosodiethylamine	0.285 (0.028)	0.276 (0.028)	0.214 (0.029)
	NS	NS	NS
NNK	0.283 (0.032)	0.319 (0.035)	0.260 (0.032)
	NS	*P* = .009	NS

NS: not statistically significant (*P* ≥ .05).

**Table 3:** Percent staining of *MKI67* in cultures exposed for 24 hours. All values expressed as percentages.

*MKI67*	Percent (%)			
	Positive small cells	Negative small cells	Positive large cells	Negative large cells
Nickel sulphate	93.6	6.3	0	20
Benzo(b)fluoranthene	92.6	7.3	0.3	19.6
N-nitrosodiethylamine	78	22	1	19
NNK	84.3	15.6	1	19

**Table 4:** Percent staining for *TP53*-positive cells.

*TP53*	Percent (%)			
	Positive small cells	Negative small cells	Positive large cells	Negative large cells
Nickel sulphate 24 hr	98.3	1.6	19	1
Nickel sulphate 48 hr	95.6	4.3	20	0
Benzo(b)fluoranthene 24 hr	68	32	19.3	0.6
Benzo(b)fluoranthene 48 hr	82.6	17.3	17.3	2.6
N-nitrosodiethylamine 24 hr	82.3	27.6	17.3	2.6
N-nitrosodiethylamine 48 hr	64.6	35.3	17.6	2.3
NNK 24 hr	92	8	17.6	2.3
NNK 48 hr	98.6	1.3	19.6	0.3

All values expressed as percentages.
