# Supplementary material for: Short-Term Exposure to Tobacco Toxins Alters Expression of Multiple Proliferation Gene Markers in Primary Human Bronchial Epithelial Cell Cultures
Source: J Oncol. 2011 Apr 5;2011:208563. doi: 10.1155/2011/208563 (PMC3137990; doi:10.1155/2011/208563)
Supplement: Supplementary file 1 — Supplemental Materials: The supplemental materials include the source of the chemical reagents, cell line, and antibodies used in this study. [file 208563.f1.pdf]

Supplemental Materials: The supplemental materials include the source of the chemical reagents, cell line, and antibodies used in this study.

Toxins: Nickel Sulphate, Cadmium Chloride, Chromium Chloride, Sodium Selenite, Polyaromatic hydrocarbons (PAH) (including Benzo[b]fluoranthene, Indeno[1,2,3-cd]pyrene, Dibenz[a,h]anthracene, 5-Methylchrysene, Dibenzo[a]pyrene, Dibenz[a,h]acridine and Benzo[k]fluoranthene), a tobacco nitrosamine (N-Nitrosodiethylamine) and Ethyl carbamate were purchased from Sigma-Aldrich (St. Louis, MO). The nicotine derivative 4-(Methylnitrosamino)-1-(3-pyridyl)-1-butanone (NNK) was purchased from Toronto Research Chemicals, Inc. (Toronto, Ontario).

Cell line: Normal human bronchial epithelial (NHBE) cells and bronchial epithelial growth medium (BEGM) BulletKit were purchased from Clonetics-BioWhittaker (San Diego, CA).

Primary antibodies: Mouse monoclonal antibodies specific to human *BIRC5*, *EGFR* and *BCL2L1* were purchased from NeoMarkers: Lab Vision Corporation (Fremont, CA); mouse monoclonal antibody specific to human *BCL-2* was purchased from Zymed Laboratories Inc. (San Francisco, CA). Mouse monoclonal antibody specific to human *MKI67*, DAB, and substrate chromogen system were purchased from DAKO North America Inc. (Carpinteria, CA). Mouse monoclonal antibody specific to human *TP53* was purchased from NovoCastra; Vision Biosystems (Norwell, Maine).
Secondary Antibody: ImmPRESS; peroxidase-conjugated anti-mouse IgG was purchased from Vector Laboratories Inc. (Burlingame, CA).

Other reagents: Phosphate buffer, sodium nitrite, methylene blue solution, pararosaniline HCL and alpha naphthyl butyrate solution were purchased from Sigma-Aldrich (St. Louis, MO). Twenty five percent glutaraldehyde solution, EM grade, was purchased from Polysciences, Inc. (Warrington, PA). Gill hematoxylin, bluing reagent and cytostain were purchased from Richard-Allan Scientific (Kalamazoo, MI). Millonigs phosphate buffer and Spurr's Kit was purchased from Electron Microscopy Sciences (Fort Washington, PA).

Dibenz[a,h]anthracene and Benzo[k]fluoranthene were excluded due to the observed solvent (toluene) cytotoxicity, the only solvent that dissolved these two carcinogens.

Trypsin/EDTA solution and trypsin neutralizing solution (Clonetics-BioWhittaker, San Diego, CA)
